# Management of hepatitis C infections among Rohingya refugees, Bangladesh

**DOI:** 10.2471/BLT.24.292920

**Published:** 2026-01-28

**Authors:** Giancarlo Ceccarelli, Francesco Branda, Fariha Fairouz, Mattia Albanese, Fabio Scarpa, Gabriella d’Ettorre, Marta Giovanetti, Terence Ngwabe Che, Massimo Ciccozzi

**Affiliations:** a Department of Public Health and Infectious Diseases, University of Rome Sapienza, Rome, Italy.; b Unit of Medical Statistics and Molecular Epidemiology, University Campus Bio-Medico, Via Álvaro del Portillo, 21, 00128, Rome, Italy.; c Dev Care Foundation, Chattogram, Bangladesh.; d Department of Biomedical Sciences, University of Sassari, Sassari, Italy.; e Sciences and Technologies for Sustainable Development and One Health, Università Campus Bio-Medico di Roma, Italy.; f World Health Organization, Cox’s Bazar, Bangladesh.

In August 2017, violence and human rights violations in Myanmar’s Rakhine State triggered a mass exodus of Rohingya refugees to Bangladesh. Large settlements were established in Cox’s Bazar, which now constitutes the world’s largest refugee camp, housing over 1 million displaced Rohingya refugees.^1^ This population faces numerous public health challenges, including malnutrition, communicable diseases, injuries, mental health disorders and a high burden of chronic noncommunicable diseases such as diabetes, hypertension and the double burden of malnutrition.^2^ The emergence and rapid increase of hepatitis C virus (HCV) infection and its sequelae, including chronic liver disease, cirrhosis and hepatocellular carcinoma is particularly concerning. 

## Hepatitis C in Rohingya camps

Epidemiological studies have documented an unexpectedly high prevalence of HCV infection among Rohingya refugees.^3^^–^^5^ Evidence from a 2023 *Médecins Sans Frontières* cross-sectional survey in Cox’s Bazar provided the most robust estimates to date. Among 641 adults randomly selected from camps in Cox’s Bazar, 30% (192/641; 95% confidence interval, CI: 26.5–34.5) were HCV-seropositive and 20% (128/641; 95% CI: 16.5–23.4) had active viraemia confirmed by polymerase chain reaction (PCR).^3^ Seropositivity was associated with female sex and prior parenteral exposures, including injections and surgical procedures. Notably, 328 (51%) respondents had never heard of hepatitis C, and only five out of 124 viraemic individuals had received prior treatment.^3^

Overcrowded conditions, unsafe syringe use, injecting drug use, unsafe blood transfusions, perinatal transmission and sociocultural practices, such as ear and nose piercing and traditional male circumcision, likely contribute to the high prevalence of hepatitis C.^6^^–^^8^ Despite preliminary studies^9^ indicating a slightly higher prevalence of the disease among the displaced population compared to the resident population, implying that when people arrived, they had already been exposed, the considerable increase in incidence suggests active and high transmission within the refugee camps. This finding underscores the need for a quick containment intervention. In response to this situation, the World Health Organization (WHO), in collaboration with the Bangladeshi government, partner agencies, international nongovernmental organizations and health experts, initiated a comprehensive HCV surveillance and treatment programme in March 2024.^4^

## The WHO programme

This programme focuses on early detection, timely treatment and prevention of mother-to-child transmission. Strategies include widespread rapid diagnostic testing, PCR confirmation for active infections and provision of pan-genotypic direct-acting antivirals, known for their high cure rates. Given the high viraemic prevalence observed, reaching up to 71% (7533/10 610) among individuals testing HCV-seropositive between October 2020 and December 2022,^3^ adopting a simplified test-and-treat approach in this setting represents a critical step towards effective disease control.

The programme’s implementing partners have established over 110 health facilities to conduct testing for hepatitis B and C in the Rohingya camps. The programme ensures that individuals who test positive are referred to 18 designated centres for further evaluation and blood sample collection, which are then sent to the WHO-supported Institute of Epidemiology Disease Control and Research field laboratory in Cox’s Bazar. These facilities coordinate confirmatory testing and treatment, ensuring systematic data collection and patient follow-up through the WHO Early Warning, Alert and Response System.^5^ To improve screening efforts, WHO has already distributed 26 000 rapid diagnostic test kits for hepatitis B virus and HCV, and is in the process of procuring an additional 50 000 rapid diagnostic test kits, together with 80 000 HCV ribonucleic acid (RNA) confirmatory test kits to enhance screening efforts.^10^ In support of the surveillance initiative, the WHO logistics team also distributed 5250 rapid diagnostic kits for cholera, human immunodeficiency virus (HIV)-syphilis, malaria and dengue, to four health-care partners serving the Rohingya community.

Furthermore, WHO has provided treatment supplies for 3900 patients diagnosed with hepatitis C and is securing additional HCV medications to treat 17 000 patients.^5^^,^^10^ As part of the hepatitis C surveillance programme, patients treated with antivirals are tested for HCV RNA levels to assess successful treatment and sustained virologic response at post-treatment week 12, which is a widely accepted efficacy endpoint for direct-acting antiviral agents. Although no data are available on HCV genotype distribution among Rohingya refugees in Cox’s Bazar, a study from Bangladesh and neighbouring Myanmar^11^ indicate a predominance of genotype 3 (including subtype 3b) and occasional detection of genotypes 1 and 6. Accordingly, the use of pan-genotypic direct-acting antiviral regimens within the WHO programme remains appropriate without requiring systematic pretreatment genotyping.

While maintaining hepatitis C screening and treatment as a priority, WHO’s integrated programme also aims to support long-term liver cancer prevention. Interventions targeting hepatitis B and C, including those designed to prevent mother-to-child transmission, seek to reduce new infections, slow disease progression and lower the risk of cirrhosis and hepatocellular carcinoma.^5^ Strengthening integrated surveillance, prevention and vaccination strategies against preventable forms of hepatitis is clearly needed in this vulnerable population, given that the prevalence of hepatitis B ranges from 12% to 18%, data on hepatitis D co-infection are lacking and exposure to hepatitis E and A viruses has been reported during jaundice outbreaks.^6^^,^^7^^,^^12^

Despite the robust design of the WHO programme, its implementation is limited by major challenges in securing adequate resources (financial, pharmacological and logistical). Overburdened health-care facilities within the refugee camps, irregular supply chains and limited access to specialized hepatology services undermine the programme’s effectiveness. At the same time, there are indications of a rising incidence of HIV,^13^ which may accelerate HCV-related liver disease progression and further complicate its management. An additional concern is the potential risk of transmission associated with injecting drug use. Although there is no definitive evidence of an epidemic driven by people who inject drugs, the issue represents an emerging challenge for the overall infection management. Given these constraints, the dynamic evaluation of adaptable strategies and treatment models is a priority to ensure that interventions remain both effective and feasible despite technical and operational challenges amid the ongoing epidemiological emergency.

## Expected outcomes 

The expected outcomes for HCV management in this vulnerable population vary considerably depending on the scale and efficacy of treatment and prevention efforts. In the most favourable scenario, universal screening and direct-acting antiviral treatment will reach the majority of infected individuals, and HCV-related morbidity and mortality could decline substantially, with cure rates exceeding 95%.^14^ This favourable scenario will also lead to a marked reduction in complications such as cirrhosis and hepatocellular carcinoma. Conversely, if systemic barriers persist and treatment coverage remains limited, a considerable proportion of the population will continue to harbour chronic HCV infections, resulting in ongoing viral transmission and a heightened risk of progressive liver fibrosis and cirrhosis. In the worst-case scenario, inadequate interventions could allow chronic HCV to persist unabated, culminating in elevated rates of cirrhosis and hepatocellular carcinoma, further straining the already fragile health-care infrastructure.

In Bangladesh, with a population of 160 million, the prevalence of HCV is less than 1%. The close contact currently occurring between Rohingyas and Bangladeshis, including intermarriage and social integration, poses a risk for the spread of the disease in the event of delayed and ineffective intervention.^6^

## Programme’s preliminary results 

In the initial phase, surveillance systems screened 4486 individuals between April 26 and May 26, 2024, revealing that 37% (1660) tested positive for hepatitis C, with 74% (1228) of those exhibiting active infection requiring treatment. These data prompted the expansion of designated centres for secondary evaluations and blood sample collection, from the current 18 to a planned total of 51 health sites.^5^

The main outcomes achieved by February 2025, one year after the project started, include the distribution of 26 000 rapid diagnostic test kits and screening of 11 275 refugees for HBV and HCV infections, and the treatment of 884 individuals. Of those treated individuals, the resulting sustained virologic response at post-treatment week 12 was around 97% (857/884).^10^

## Addressing needs

While WHO’s programme in Cox’s Bazar is an important step towards mitigating the burden of hepatitis C among Rohingya refugees, its long-term success depends on addressing operational challenges and securing the needed resources. Researchers estimate that the cost of screening and treating the entire refugee population would cost approximately 37 million United States dollars (US$).^4^

Although the WHO programme demonstrates promising early results, its sustainability is jeopardized by the current financing challenges and the high prevalence observed over time. For instance, the European Union has recently only committed 1 million euros in humanitarian aid to enhance hepatitis C efforts in Cox’s Bazar, while Japan has pledged US$ 1.6 million to support essential health-care services, focusing on the diagnosis and treatment of hepatitis C for Rohingya refugees.^10^^,^^15^ On the other hand, the withdrawal of United States of America from WHO and a concurrent freeze on the United States’s humanitarian and development aid have introduced additional uncertainty, potentially undermining funding stability. From an epidemiological and economic standpoint, these funding fluctuations may result in inconsistent programme implementation, jeopardizing long-term public health outcomes. Without robust and continuous investment, the cost of unmanaged HCV, in terms of increased health-care expenditure due to liver failure, cirrhosis and hepatocellular carcinoma, could far exceed the savings achieved through early intervention. Both national and international stakeholders should prioritize sustained financial support, adequate diagnostic and treatment supplies, and the operational capacity needed to ensure the success of HCV virus management initiatives in this complex humanitarian context. 
